# Supporting child witnesses during identification lineups: Exploring the effectiveness of registered intermediaries

**DOI:** 10.1002/acp.3412

**Published:** 2018-04-02

**Authors:** Rachel Wilcock, Laura Crane, Zoe Hobson, Gilly Nash, Mimi Kirke‐Smith, Lucy A. Henry

**Affiliations:** ^1^ University of Winchester Winchester UK; ^2^ UCL Institute of Education University College London London UK; ^3^ Mayor's Office for Policing and Crime London UK; ^4^ City, University of London London UK

**Keywords:** identification, intermediaries, child witnesses

## Abstract

Performance at identification lineup was assessed in eighty‐five 6‐ to 11‐year‐old typically developing children. Children viewed a live staged event involving 2 male actors, and were asked to identify the perpetrators from 2 separate lineups (one perpetrator‐present lineup and one perpetrator‐absent lineup). Half the children took part in lineups adapted by a registered intermediary (an impartial, trained professional who facilitates understanding and communication between vulnerable witnesses and members of the justice system), and half took part in “best‐practice” lineups, according to the current guidance for eyewitness identification in England and Wales. Children receiving assistance from a registered intermediary (relative to children who received best‐practice lineups) were more accurate in their identifications for perpetrator‐present lineups, and there was some evidence that they were also more accurate for perpetrator‐absent lineups. This provides the first empirical evidence for the effectiveness of registered intermediary support during identification lineups.

## 1 INTRODUCTION

Despite a long tradition of research on eyewitness identification abilities of adults, less attention has been paid to the capabilities of child witnesses (Rush et al., 2014). A recent meta‐analysis (of 20,000 participants across 91 studies) concluded that child witnesses (and older adults) were less accurate than young adults when taking part in identification lineups (Fitzgerald & Price, 2015). Specifically, child witnesses were less likely to correctly identify perpetrators when they were present in the lineup (contrary to previous reports, e.g., Pozzulo & Lindsay, 1998), and were more likely to erroneously select an innocent “foil” when the perpetrator was not present in the lineup (Fitzgerald & Price, 2015).

The demands of considering and comparing six or more separate images of faces in sequentially presented identification lineups are considerable. Face recognition, working memory, and executive functions may all be required, with working memory demands being particularly acute because working memory may have a maximum capacity of three to five chunks of information (Cowan, 2010). Further, these component skills improve markedly during childhood (e.g., Bruce et al., 2000; Diamond, 2013; Henry, 2012), making it imperative to put in place effective procedures to best support vulnerable child witnesses.

Adaptations to lineup procedures (particularly for perpetrator‐absent lineups) have been suggested to make them more appropriate for child witnesses (Fitzgerald, Whiting, Therrien, & Price, 2014). Pozzulo and Lindsay (1999) proposed “elimination lineups” to reduce the rate of false positive responding (i.e., selecting an innocent foil from a perpetrator‐absent lineup). This requires children to identify the person from the lineup that they think is the most similar in appearance to the perpetrator, before asking them to decide whether that person is, in fact, the perpetrator. This reduces the likelihood of children using a relative decision making strategy when viewing a simultaneous lineup, which may lead to an increase in false identifications. With 10‐ to 14‐year‐old children, Pozzulo and Lindsay (1999) found this technique to decrease false positive responding without affecting performance on perpetrator‐present lineups. Similarly, Zajac and Karageorge (2009) suggested including a “wildcard”—an additional photograph of a silhouette superimposed with a question mark—to lineups, asking children to point to the wildcard if the perpetrator is *not* present. The wildcard serves as a reminder that the perpetrator may or may not be present and may reduce the chance of false identifications. This technique did improve 8‐ to 11‐year‐old children's accuracy in perpetrator‐absent lineups, without having a concomitant effect on correct identifications in perpetrator‐present lineups (Zajac & Karageorge, 2009). Although promising, neither of these techniques have been implemented in best‐practice guidelines for lineup identification in England and Wales.

In England and Wales, vulnerable witnesses (including children) are entitled to a registered intermediary (RI) at all stages of an investigation (e.g., during interview, identification lineup, and trial). An RI is an impartial, trained professional who facilitates understanding and communication between vulnerable witnesses and members of the justice system; ensuring that communication is complete, coherent, and accurate (Ministry of Justice, 2015). The RI role was developed as part of the range of “special measures” introduced for cases involving victims and vulnerable witnesses (Youth Justice & Criminal Evidence Act; YJCEA, 1999), and the use of RIs has been steadily increasing since the introduction of the Witness Intermediary Scheme pilot project in 2004 (Home Office National Crime Agency, personal communication).

The role of RIs is wide‐ranging, but includes conducting an initial assessment of the witness (including his/her language and communication skills); preparing reports detailing recommendations at different stages of the justice process; and advising more widely on how to enable the vulnerable individual to communicate their best evidence (Plotnikoff & Woolfson, 2015). From time to time, an RI will be required to assist during a PROMAT™ video recorded identification parade. The RI's initial assessment will inform which strategies are recommended for the vulnerable witness to engage with the identification process. However, as there is no set procedure or template for an RI assessment, its form and content—and the subsequent strategies recommended—will depend on both the witness's communication needs and the expertise and specialism of the RI (Ministry of Justice, 2015).

Evaluations of the Witness Intermediary Scheme have been positive. Discussions with witnesses and their families, as well as legal professionals (e.g., police officers, judges, and advocates), suggest that the use of RIs is associated with increased access to justice, at both investigative and trial stages of cases (Henderson, 2015; Plotnikoff & Woolfson, 2007, 2015). More recently, Henry, Crane, et al. (2017) conducted an experimental study exploring children's recall of a staged event, in which the assistance of an RI was compared against a “best‐practice” police interview. Here, typically developing 6‐ to 11‐year‐old children recalled more correct information, without a decrease in accuracy (relative to the best‐practice police interview), when provided with an RI.

The current paper presents additional novel data from this investigation, focusing on RI intervention during identification lineups. This represents for the first time that RI assistance during identification lineups has been evaluated and, therefore, has important implications for practice. In the current study, children watched a staged event involving a mock crime. They gave an initial brief account of what they saw immediately after the event (akin to a statement given to a response police officer) and, 1 week later, took part in a full evidential interview and identification lineup (at which some of the children received the support of an RI). The RI intervention at lineup included recommended adaptations to Police and Criminal Evidence Act (1984) Code D practice: showing the sequential lineup presentation once, opposed to twice, which could reduce fatigue and memory decay by reducing the presentation period; showing a simultaneous matrix of faces from the lineup, which could decrease the working memory load (Cowan, 2010) and lead to an improvement in lineup performance on both perpetrator present and absent lineups; and emphasising non‐biased lineup instructions to highlight that the perpetrator may or may not be present, which could lead to reduced rates of false choosing on perpetrator absent lineups (Malpass & Devine, 1981). A verbal description of the format of the identification lineup was also given at an age appropriate pace before the task began. Given the lack of previous research evidence, predictions were tentative. However, it was expected that children supported by an RI—given the reduction in cognitive demands and the emphasising of nonbiased lineup instructions—would show greater lineup accuracy.

## 2 METHOD

### 2.1 Participants

Participants were English‐speaking 6‐ to 11‐year‐old typically developing children attending one of four mainstream primary schools based in low/mid SES areas in a large, multi‐ethnic city (Greater London, UK). The sample comprised 85 children (41 boys; 44 girls) between the ages of 6 years 6 months and 11 years 2 months (mean = 8 years 6 months), none of whom had diagnosed developmental disorders or special educational needs. The age range was selected as it encompassed a range of ages utilised in previous research, but was restricted enough to ensure that the staged event was suitable for all participating children. Participants were semirandomly allocated to the RI or best‐practice condition; strict random allocation was impossible due to practical issues, schools, and the need to test all children in the RI condition last (to prevent cross‐fertilisation to our interviewers, see Henry, Crane, et al., 2017, for details).

#### 2.1.1 Power analysis

In the RI condition, there were 19 participants per cell, and in the best‐practice condition, there were between 20 and 27 participants per cell, which is consistent with the norm within the eyewitness identification literature of including approximately 20 participants per cell. For a chi‐square examining the effect of the main independent variable (RI/best practice) on lineup accuracy (correct/incorrect), a post hoc power analysis on the sample of 85 was conducted using the software package, GPower (Faul, Erdfelder, Lang, & Buchner, 2007). The recommended effect sizes used for this assessment were as follows: small (*w* = .10), medium (*w* = .30), and large (*w* = .50; see Cohen, 1988). The alpha level used for this analysis was *p* < .05. Post hoc analyses revealed that the statistical power for this study was .15 for detecting a small effect, .79 for detecting a medium effect, and in excess of .99 for detecting a large effect. Thus, there was adequate power at the medium effect size and more than adequate power to detect a large effect size, but less than adequate statistical power to detect a small effect size.

### 2.2 Materials and procedure

#### 2.2.1 Event and brief interviews

Children watched a live event during school assembly of two male actors giving a talk about what school was like a long time ago. Towards the end of the talk, a minor crime (the theft of keys/phone) took place (see Henry, Crane, et al., 2017, for full details). Although children were randomly assigned to one of two parallel talks (each involving slightly different materials and different names for the key actors), the actors were the same across both versions and there was no significant effect of “event version” on lineup accuracy 1Accurate = correct identifications of the perpetrator and correct rejections of a perpetrator absent lineup; inaccurate = foil identifications or false identifications and incorrect rejections of a perpetrator present lineup. for Perpetrator 1, χ^2^(1, *N* = 85) = 1.07, *p* = .30, or Perpetrator 2, χ^2^(1, *N* = 85) = .16, *p* = .69. Data for the two events were, therefore, combined.

Immediately after viewing the event, all children were questioned about what they saw; akin to a statement being taken from a response officer (see Henry, Messer, et al., 2017, for data concerning these “brief interviews”). The Brief Interview used a standardised protocol beginning with an open question “tell me what you remember about what you just saw” and a series of follow‐up prompts (who was there, what did they do, what did they look like, when did it happen, where did it happen) that could be used depending upon the children's response to the open question. Although almost all children referred to the perpetrators within their interviews, descriptions tended to focus on clothing and hair, rather than facial features.

#### 2.2.2 Investigative interviews

One week later, children took part in one of four investigative interviews (see Henry, Crane, et al., 2017, for full data concerning the “investigative interviews”), but it is the best‐practice police interview and the RI‐assisted interview that are relevant to the current paper. The best‐practice interview was based on Achieving Best Evidence principles (Home Office, 2011) and had seven discrete phases: (a) greet and personalise the interview; (b) rapport building (general chit‐chat with the child); (c) truth and lies exercise (e.g., determining whether the child correctly responds to a statement along the lines of “that lady is wearing a blue jumper” when it is red); (d) explain the purpose of the interview; (e) free recall (recall Attempt 1—“Tell me everything you can remember about what you saw”); (f) questioning (recall Attempt 2—using open questions based upon what the child had already recalled); and (g) closure. As per the brief interviews, most children referred to the perpetrators in their interviews, but descriptions tended to focus on hair and clothing (opposed to facial features).

Children in the RI condition were individually assessed prior to their interview (as advised by Plotnikoff & Woolfson, 2015). This assessment—conducted in both the classroom and in a face‐to‐face assessment—included general rapport building; assessments of the child's communication abilities in various areas (e.g., ability to talk about past events, comprehension, and understanding); and an assessment of the child's needs and abilities regarding additional concrete or visual communication aids (paper and pens, generic small world figures and furniture), full details of which are presented in Henry, Crane, et al. (2017). Based on the assessment, RIs provided written and verbal recommendations to the interviewer and lineup administrator (trained postdoctoral research assistants) for all aspects of the interview and identification lineup. As this was a sample of typically developing children (with no communication or special educational needs), the adaptations for identification lineups applied to all children (see key recommendations below). These largely involved simplifying procedures and word/sentence structure to make instructions age appropriate (given that the task the children were undertaking was unfamiliar to them). There was a meeting between the RI and the interviewer/lineup administrator before each child's interview to discuss the recommendations, during which the RIs flagged any individual needs (e.g., that a child may have poorer attention than another child and might benefit from information being provided at a slower pace or in smaller chunks). Children were provided with no visual details (or photographs) of the actors in the scene during any of the interviews, even in the RI condition, although some RI interviews did incorporate small world characters that were generic in appearance. Importantly, RIs have Codes of Practice and of Ethics (see Ministry of Justice, 2015), and must work within what is legally acceptable to the courts—not making any recommendations that could jeopardise a fair trial. Their role is to facilitate communication during investigation and trial within acceptable boundaries and these were the principles followed in the current research.

The two RIs involved in this study each had over 10 years of experience, including with typically developing children of the ages included in this research (6–11 years). They had completed necessary training provided by the Ministry of Justice, and also contributed to this training course for several cohorts of new RIs.

#### 2.2.3 Identification lineups

Immediately following the investigative interviews, children viewed two video lineups; one for each actor in the staged event. Each child viewed one “perpetrator present” lineup (in which one of the actors was present) and one “perpetrator absent” lineup (in which neither actor was present). Some children (*n* = 42) saw Perpetrator 1 in the first lineup and other children (*n* = 43) saw Perpetrator 1 in the second lineup, and vice versa. There was no effect of lineup order for Perpetrator 1 accuracy, χ^2^(1, *N* = 85) = .17, *p* = .68, or Perpetrator 2 accuracy, χ^2^(1, *N* = 85) = .30, *p* = .59, so data were combined across the lineups.

The lineups were produced by the UK's Metropolitan Police Service in accordance with Police and Criminal Evidence Act (PACE, 1984) Code D (2011), which gives provision for identification procedures in England and Wales. Each lineup contained nine (colour) video images of head and shoulders, facing front. Heads turned to the left and to the right profile, and then back to the front. PACE Code D specifies that each video “lineup” consists of a minimum of nine images, including one suspect; the witness must be advised that the culprit may not be present; the witness must view the entire sequential lineup twice before making any identification; and if the witness is unable to make a positive identification they should say so. The foils contained within each lineup were chosen by experienced police employees using their national database, PROMAT™. The lineups for the children in the best‐practice condition were run in accordance with PACE Code D (as described below). For logistical reasons, lineup administrators were not blind to the identity of the perpetrator, but they sat behind each child out of his/her eye line to avoid inadvertently influencing the lineup result.

The possible lineup responses were correct hits of the perpetrator, foil identification, or incorrect rejection (for perpetrator present lineups); or correct rejections, false identification of innocent suspect, or foil identification (for perpetrator absent lineups). To ensure the lineup was not biased towards the suspect, a measure of lineup bias was calculated (Malpass & Lindsay, 1999). For Perpetrator 1, 4 of 30 mock witnesses (.13) chose the perpetrator, and for Perpetrator 2, 5 of 30 mock witnesses chose the perpetrator (.16); both of which are only slightly higher than what would be expected by chance (.11). Similar results were also found for the innocent suspect replacing Perpetrator 1 (.10) and the innocent suspect replacing Perpetrator 2 (.16) in the perpetrator absent lineups. These results suggest that the lineups were not biased toward the perpetrators or innocent replacements.

The registered intermediary Procedural Guidance Manual (Ministry of Justice, 2015) confirms that RIs may assist witnesses taking part in identification procedures. The nature of this “assistance” is not explicitly outlined, but the purpose of the RI role is to allow vulnerable witnesses to give their best evidence (Ministry of Justice, 2015). As such, the RIs in this study provided advice on how to adapt the identification lineup instructions and procedure, making five key recommendations. These recommendations were based on the RI's understanding of typical communication skills of 6‐ to 11‐year‐olds and had been approved as within the guidelines of PACE Code D (2011) in two real cases (involving one of the RIs in this study) prior to the development of the research protocol. The recommendations were considered appropriate for all children in the study, being general enough to be useful for the range of ages and abilities included. The recommendations were that the “lineup administrator” (one of four trained postdoctoral research assistants) would (a) show children the series of nine video images sequentially once (opposed to twice, as per PACE Code D guidance); (b) then show all nine images simultaneously in a static photo matrix (note that although there is provision for this in PACE Code D, this is not part of the standard procedure); (c) provide several different response options to the children (including spoken, written, and visual aids), stating that if they recognised one of the people, they could (1) tell the RI “I see the person,” (2) tell the RI, or write down, the number of the person (each image was assigned a unique number from 1 to 9), or (3) point to the face; (d) tell children that if they did not see one of the people, they could (1) tell the RI “I can't see the person” or (2) point to a card with a red cross that was placed on the table; and (e) the RIs checked the children's understanding of their lineup responses both verbally and pictorially [children could either choose a “thumbs up” picture, suggesting they thought their lineup decision was correct; a picture of a person looking unsure, suggesting they did not know if their lineup decision was correct; or a “thumbs down” picture, suggesting they thought their lineup decision could be wrong]. Note that despite the inclusion of this “checking understanding” task, we took only the *original* lineup response as the child's answer, in accordance with traditional confidence judgement procedures commonly used in the eyewitness literature (see Wilcock, Bull, & Milne, 2008).

Importantly, although the RIs were present throughout the lineups (seated next to the child), their role did not extend beyond giving advice to the lineup administrator regarding instructions and procedure, as listed above. In addition, RIs had no knowledge of the identity of the perpetrators. There were two RIs involved in the study. For Perpetrator 1, one RI (RI2) elicited greater lineup accuracy than the other (RI1), χ^2^(1, *N* = 38) = 5.76, *p* = .02. For Perpetrator 2, there was no significant effect of RI on lineup accuracy, χ^2^(1, *N* = 38) = .00, *p* = 1.00. Inspection of the data collected from the children seen by each RI (*n* = 19 each) revealed that the children were comparable across all control measures (discussed next), although there was a trend towards the children seen by RI2 having higher facial memory scaled scores (mean = 12.89, *SD* = 2.75) on a subtest of a standardised memory battery (the Test of Memory and Learning 2; Reynolds & Voress, 2007) than the children seen by RI1 (mean = 11.11, *SD* = 3.00), *t*(36) = −1.92, *p* = .06. This may account for the better performance of the children seen by RI2, relative to RI1, on one of the lineups.

#### 2.2.4 Control measures

An extensive range of standardised cognitive measures (intelligence, language, memory, and attention) were administered to ensure that cognitive skills that might affect identification lineup performance were controlled between groups (see Anderson, Carlson, Carlson, & Gronlund, 2014; Wilcock et al., 2008). Table 1 includes details about age, IQ, language, memory, and attention variables (all suitable for the age ranges tested in this study) that were assessed.

**Table 1:** Mean scores (standard deviations) on background variables for children in each interview condition

Variables:	Best practice (*n* = 47, 21 girls)	Registered intermediary (*n* = 38, 23 girls)	Group differences
Age	8 years 2 months (13 m)	9 years 1 month (16 m)	*p = .001
WASI‐II^a^	103.7 (12.2)	102.5 (14.3)	n.s.
TOMAL2 composite^a^	108.2 (16.7)	110.1 (16.4)	n.s.
TOMAL2 verbal^a^	108.4 (17.7)	106.5 (16.6)	n.s.
TOMAL2 non‐verbal^a^	106.3 (19.3)	111.6 (20.0)	n.s.
TOMAL2 facial memory^b^	10.1 (3.6)	12.0 (3.0)	*p = .01
BPVS‐3^a^	90.5 (12.5)	87.9 (14.9)	n.s.
ELT‐2 sequencing^a^	107.5 (10.6)	109.4 (6.5)	n.s.
ELT‐2 grammar & syntax^a^	106.2 (12.3)	103.7 (10.8)	n.s.
CELF‐4 recalling sentences^b^	9.7 (3.2)	10.8 (3.1)	n.s
CELF‐4 formulated sentences^b^	9.1(3.4)	9.1 (3.2)	n.s.
TEA‐Ch sky search^b^	9.3 (2.6)	9.2 (3.3)	n.s.
TEA‐Ch score!^b^	8.5 (3.1)	9.3 (3.6)	n.s.
TEA‐Ch dual task^b^	6.2 (4.0)	5.3 (3.6)	n.s.

*Note*. BPVS‐3 = British Picture Vocabulary Scale third edition; CELF‐4 UK= Clinical Evaluation of Language Fundamentals, UK 4th edition; ELT‐2 = Expressive Language Test 2; TEA‐Ch = Test of Everyday Attention for Children; TOMAL2 = Test of Memory and Learning 2; WASI‐II = second edition of the Wechsler Abbreviated Scale of Intelligence.

a Standardised scores (mean = 100, *SD* = 15);

b Scaled scores (mean = 10, *SD* = 3).

##### Intelligence

Two subtests (“vocabulary” and “matrix reasoning”) from the second edition of the Wechsler Abbreviated Scale of Intelligence (Wechsler & Zhou, 2011) were used to establish suitability for the study, and to provide an assessment of verbal, non‐verbal, and full‐scale IQ.

##### Language

Although identification lineups involve visual identification of a perpetrator, it is important that the child witness understands the instructions and procedure. As such, several receptive and expressive language measures were included: the British Picture Vocabulary Scale Third Edition (Dunn, Dunn, & Styles, 2009); two subtests (“sequencing,” “grammar and syntax”) of the Expressive Language Test 2 (Bowers, Huisingh, LoGiudice, & Orman, 2010); and two subtests (“recalling sentences,” “formulating sentences”) of the Clinical Evaluation of Language Fundamentals, UK, 4th edition (Semel, Wiig, & Secord, 2006).

##### Memory

Four of the eight core subtests from the Test of Memory and Learning 2 (Reynolds & Voress, 2007) were used to provide a composite memory measure reflecting both verbal memory (“memory for stories” and “paired recall”) and non‐verbal memory (“facial memory” and “visual sequential memory”) memory. As facial memory was of particular interest, scores on this subtest are reported separately.

##### Attention

The Test of Everyday Attention for Children (Tea‐Ch; Manly, Robertson, Anderson, & Nimmo‐Smith, 1999) was used to assess a range of attention skills relevant to identification lineups, including selective/focused attention (the “sky search” subtest), sustained attention (the “Score!” subtest) and sustained‐divided attention (the “sky search dual task” subtest).

## 3 RESULTS

First, potential group differences in cognitive variables that might impact on witness performance were assessed (age, IQ, language, memory, and attention). Table 1 includes mean ages for participants in each condition and standardised/scaled scores (and *SD*s) on all cognitive variables. Age differed between the two groups, as did facial memory. Therefore, these variables were controlled in subsequent analyses.

To examine the effect of condition (RI vs. best‐practice) on lineup accuracy and perpetrator presence or absence, whilst controlling for variables that differed between groups (age, facial memory), two logistic regressions were conducted (one for each perpetrator). For both perpetrators, logistic regression analyses were performed with lineup accuracy (correct or incorrect) as the dependent variable. Predictor variables were condition, lineup presence, facial memory, and age in months.

For Perpetrator 1, all cases (*n* = 85) were analysed and the full model significantly predicted lineup accuracy (omnibus χ^2^ = 13.87, *df* = 4, *p* = .008). The model accounted for between 15.1% and 21.6% of the variance, with 82% of correct performance (but only 62.5% of incorrect performance) successfully predicted. Table 2 provides coefficients, the Wald statistic, associated degrees of freedom, and probability values for each of the predictor variables. These illustrate that only condition and perpetrator presence reliably predicted lineup accuracy. The odds ratio of an accurate lineup response occurring on the best‐practice lineup was 1.47, whereas it was 6.6 on the RI lineup; thus, participants in the RI condition were more likely to be correct. The odds ratio of an accurate lineup response occurring on the perpetrator present lineup was 1.56, whereas it was 5.5 on the perpetrator absent lineup; therefore, participants were more likely to be correct on the perpetrator absent lineup. Follow‐up chi‐squared analysis (or Fisher's exact test where expected frequencies were less than 5) were conducted to examine the effect of condition on perpetrator present and absent lineup accuracy, respectively. On the perpetrator present lineup, the presence of an RI led to a higher number of correct identifications, χ^2^(1, *n* = 46) = 7.40, *p* = .007, than the best‐practice condition (see Table 3). The odds ratio of an accurate lineup response occurring on the best‐practice perpetrator present lineup was .8 and on the RI perpetrator‐present lineup was 5.33; thus, participants were more likely to be correct when in the RI condition. On the perpetrator absent lineup, Fisher's exact test revealed no significant effect of condition on lineup accuracy *p* = .66.

**Table 2:** Logistic regression predictors for Perpetrator 1 and 2 accuracy

	Perpetrator 1							Perpetrator 2						
Predictors	*B*	Wald	Degrees of freedom	Significance	*Exp B*	95% confidence interval		*B*	Wald	Degrees of freedom	Significance	*Exp B*	95% confidence interval	
						Lower	Upper						Lower	Upper
Condition	1.44	5.11	1	.02	4.21	1.21	14.67	2.17	10.19	1	.001	8.74	2.31	33.06
Target presence	1.27	5.17	1	.02	3.57	1.19	10.71	−.97	3.12	1	.08	.38	.13	1.11
Age	−.00	.04	1	.84	1.00	.96	1.04	.008	.16	1	.69	.99	.96	1.03
Facial memory	−.01	.02	1	.88	.99	.85	1.15	.09	1.34	1	.25	1.10	.94	1.28

*Note. N* = 85.

**Table 3:** Identification performance for Perpetrator 1 and Perpetrator 2 by condition and perpetrator presence

	*n*	Perpetrator 1						Perpetrator 2					
		Perpetrator‐present			Perpetrator‐absent			Perpetrator‐present			Perpetrator‐absent		
		Hit (%)	Foil ID (%)	Incorrect rejection (%)	Correct rejection (%)	Foil ID (%)	False ID (%)	Hit (%)	Foil ID (%)	Incorrect rejection (%)	Correct rejection (%)	Foil ID (%)	False ID (%)
Best practice	47	44 (12)	30 (8)	26 (7)	80 (16)	15 (3)	5 (1)	60 (12)	30 (6)	10 (2)	48 (13)	52 (14)	0 (0)
Registered intermediary	38	84 (16)	11 (2)	5 (1)	89 (17)	5 (1)	5 (1)	100 (19)	0 (0)	0 (0)	79 (15)	16 (3)	5 (1)

*Note. N* = 85.

For Perpetrator 2, again, all cases (*n* = 85) were analysed and the full model significantly predicted lineup accuracy (omnibus *χ* ^*2*^ = 18.70, *df* = 4, *p* = .001). The model accounted for between 19.7% and 27.9% of the variance, with 84.7% of correct performance (but only 42.3% of incorrect performance) successfully predicted. Table 2 gives coefficients, the Wald statistic, associated degrees of freedom, and probability values for each of the predictor variables. These illustrate that only condition reliably predicted lineup accuracy. The odds ratio of an accurate lineup response occurring on best‐practice lineups was 1.14, and on RI lineups was 8.5; participants were more likely to be correct when in the RI condition. Follow‐up chi‐squared analysis (or Fisher's exact test where expected frequencies were less than 5) to examine the effect of condition on accuracy for perpetrator present and absent lineups, respectively, demonstrated a significant effect of condition. RI presence led to a greater number of correct identifications on the perpetrator present lineup (Fisher's exact test *p* = .003) compared with the best‐practice condition. The odds ratio of an accurate lineup response occurring on the best‐practice perpetrator present lineup was 1.5, whereas on the RI perpetrator present lineup, all 19 participants were correct. RI presence led to a greater number of correct rejections on the perpetrator absent lineup, χ^2^(1, *n* = 46) = 4.44, *p* = .04, compared with the best‐practice condition (see Table 3). The odds ratio of an accurate lineup response occurring on the best‐practice perpetrator‐absent lineup was .93, and on the RI perpetrator absent lineup was 3.75.

As previously noted, scores on the facial memory subset of the Test of Memory and Learning differed between children in the two conditions, with children in the RI condition performing better on this subtest than the children in the best‐practice condition. Because facial memory is likely to be closely linked to identification performance, further analyses were conducted after removing nine children with the highest facial memory scores from the RI group, in order to check whether the significant effect of condition remained when facial memory scores were equivalent, *t*(74) = −1.08, *p* = .29. The results remained the same: for both perpetrators, there was a significant effect of condition on lineup accuracy: Perpetrator 1, χ^2^(1, *n* = 76) = 4.46, *p* = .04; and Perpetrator 2, χ^2^(1, *n* = 76) = 13.22, *p* = .001. For Perpetrator 1, the odds ratio of an accurate lineup response occurring on the best‐practice lineup was 1.47 and on the RI lineup was 4.8. For Perpetrator 2, the odds ratio of an accurate lineup response occurring on the best‐practice lineup was 1.14 and on the RI lineup was 13.5; thus, participants were more likely to be correct when in the RI condition for both perpetrators.

## 4 DISCUSSION

Although the use of RIs in England and Wales is steadily increasing, there has been little empirical work exploring their efficacy, and none concerning their potential impact on identification lineups. This study was a novel evaluation of the effectiveness of RI assistance during an identification lineup in 6‐ to 11‐year‐old typically developing children. Results demonstrated a beneficial effect of RI intervention on lineup identification accuracy: adaptations to the lineup protocol suggested by RIs resulted in a higher number of correct identifications for perpetrator present lineups, with some evidence that these adaptations were also effective for perpetrator absent lineups.

The current findings have forensic relevance in jurisdictions worldwide, given the international interest in RI schemes (Cooper & Wurtzel, 2014; Plotnikoff & Woolfson, 2015), as well as the anecdotal (see Plotnikoff & Woolfson, 2015) and experimental (see Henry, Crane, et al., 2017) evidence for their effectiveness. However, existing evidence largely focuses on the role of RIs during investigative interviews or court proceedings. The current research represents the first empirical study to explore the effect of RIs on the accuracy of children's identifications. It is important to consider why the findings were positive.

The RIs introduced five key recommendations to the identification procedure. First, they adapted the way the lineup was presented: only showing the children the lineup sequentially once (opposed to twice, as per PACE Code D). This could have been effective via reducing fatigue (which may be likely in child witnesses). Additional analyses were conducted to explore this explanation (see Appendix A), demonstrating that there were no differences in lineup accuracy for the first and second lineups given to children in the RI condition; perhaps unsurprising given that the lineup procedure was relatively short. However, there was also no difference in lineup accuracy between the first and second lineups for children in the best‐practice condition; a condition in which the procedure was significantly longer and where one might expect to see effects of fatigue. As such, this explanation seems unlikely.

A second RI recommendation was that, following the single sequential viewing of the lineup, children were shown a simultaneous matrix of all members of the lineup. Although there is provision in PACE Code D to do this if a witness requests it, it is not routinely offered. This could have led to more correct identifications in the RI condition by reducing the working memory load inherent in comparing nine separate images consecutively (Cowan, 2010), particularly when the delay periods exceed short‐term memory limits. There has been controversy regarding lineup presentation methods, because data show different outcomes across perpetrator present and absent lineups. Sequential (as opposed to simultaneous) lineup presentation is likely to lead to a reduction in foil identifications and more correct rejections from perpetrator absent lineups. However, a simultaneous presentation is more likely to elicit correct identifications of the perpetrator compared with a sequential presentation (Steblay, Dysart, & Wells, 2011). Here, sequential followed by simultaneous lineup presentation may have allowed witnesses in the RI condition to first encode all faces individually and then compare them simultaneously without a working memory load. Indeed, Lindsay, Lea, and Fulford (1991; Experiment 1) found that allowing the witness to view a lineup simultaneously after initially viewing it sequentially lead to a small increase in the rate of correct identifications from a perpetrator present lineup, mirroring the findings presented here in the RI condition (although see Wilcock & Kneller, 2011, for conflicting results).

A further change the RIs made was simplifying the instructions and procedures of the lineup, making them more appropriate for 6‐ to 11‐year‐old children (Recommendations 3 to 5). In PACE Code D, there is no protocol for adapting the lineup for vulnerable child witnesses, however, in this study, RIs highlighted the different lineup response options (verbally and pictorially—Recommendations 3 and 4) and checked the child's understanding of their response (again, verbally and pictorially—Recommendation 5). Previous research has shown that using visual prompts during a lineup (e.g., a wildcard that children can point at to indicate the person is not present) can be effective at improving lineup performance (Zajac & Karageorge, 2009). This serves to remind the children of the range of response options available to them (which were explained in the initial lineup instructions, but may have been forgotten by the time they were asked to provide a response).

Overall, sequential (followed by simultaneous) lineup presentation, plus the simplification of the instructions and procedures (e.g., the option of visual responding), could have been key features that improved performance for children supported by an RI. Further research is required to replicate these findings and to tease out which RI adjustments are helpful to child witnesses.

In this study, the presence of an RI improved performance on both perpetrator present lineups, but only had a positive effect on one of the two perpetrator absent lineups. Previous research has shown that children find perpetrator absent lineups more problematic than perpetrator present lineups. This could be because the mere presentation of photos/videos could imply to the child that the perpetrator features in the lineup, and that they are required to make a selection (Zajac & Karageorge, 2009). For children in the best‐practice condition, there was a reduced hit rate for Perpetrator 1 compared with Perpetrator 2. This could suggest that the lineup for Perpetrator 1 was slightly more difficult. If this was the case for the perpetrator absent lineup, increased lineup difficulty could lead to children not being able to make a selection, thus resulting in an increased rate of correct rejections for Perpetrator 1, regardless of presence or absence of the RI.

Currently, there are no official guidelines regarding how RIs can make adaptations to lineup protocols. This is because most RI work focuses on the investigative interview or trial stages of a criminal case, with little consideration given to the RI role during identification lineups. In this study, the two experienced, practising RIs developed a protocol for the lineups, based on their knowledge and experience of typically developing children of this age and one RI's previous experience of assisting during real life identification lineups. It is important that the RI role during identification lineups is formalised. Should an RI be asked to assist with an identification lineup, it will be essential for them to liaise with the lineup administrator to determine which strategies can be implemented to best support the vulnerable witness (akin to a Ground Rules Hearing, in which judges in England and Wales can outline the directions necessary to ensure that vulnerable individuals can fairly and effectively participate in court proceedings; see Cooper, Backen, & Marchant, 2015, for information). Further, lineup administrators should receive training on the purpose and nature of the RI role, to ensure that professionals work together effectively, to best meet the needs of the vulnerable witness.

In conclusion, this study highlights beneficial effects of RIs during identification lineups with 6‐ to 11‐year‐old typically developing children. However, one limitation is that this was an experimental study involving a mild and nonthreatening staged event. In addition, by the time the children were engaged in the lineup process (which was conducted in a familiar environment), they were comfortable with the RI and the interviewer/lineup administrator (having worked with them on several occasions). In real‐life, children may be taking part in a lineup identification whilst experiencing high levels of stress and anxiety, which may (negatively) impact on their performance. One crucial aspect of the RI role is to assist in the management of such issues: to enable any trauma to be managed appropriately and to ensure the witness can engage and communicate with justice professionals (The Advocate's Gateway, 2015). As such, it is possible that we have *under*estimated the beneficial effects of RIs in this study. Nevertheless, there were several positive features of this study, including the fact that the children were shown a live event (opposed to a video) and there was a realistic 1‐week delay between the presentation of the staged event and the subsequent lineup. As such, the research was more ecologically valid than many other studies on the performance of child witnesses during lineups, giving confidence in the results.

## APPENDIX A.

### A..1

One key change that the registered intermediaries (RIs) made to the presentation of the lineups was to show the series of nine video images sequentially once, opposed to twice (as per PACE Code D guidance). Then, following the single sequential presentation, children in the RI condition viewed all nine images simultaneously in a static photo matrix. Hence, the procedure for children in the RI condition was quicker than for those in the best‐practice condition. As a longer lineup duration may lead to fatigue effects in child witnesses, data were analysed to explore lineup accuracy on the first lineup the children viewed (henceforth, “Lineup 1”) and the second lineup (henceforth “Lineup 2”). Lineup accuracy was not affected by which of the two actors was featured in the lineup, and this was true for both Lineup 1, χ^2^(1, *N* = 85) = .19, *p* = .67, and Lineup 2, χ^2^(1, *N* = 85) = .09, *p* = .76, therefore, data for the two actors were combined (see Table 3). McNemar tests were conducted to examine whether children were more accurate on Lineup 1 or Lineup 2 and this revealed no significant effect of Lineup, *N* = 85, *p* = .42. This was true for children in the best‐practice condition, *n* = 47, *p* = .63, as well as those in the RI condition, *n* = 38, *p* = .73, suggesting fatigue effects were not present.
